# A Reduction of Calcineurin Inhibitors May Improve Survival in Patients with De Novo Colorectal Cancer after Liver Transplantation

**DOI:** 10.3390/medicina58121755

**Published:** 2022-11-29

**Authors:** Ramin Raul Ossami Saidy, Elisa Wegener, Deniz Uluk, Luca Dittrich, Wenzel Schöning, Georg Lurje, Robert Öllinger, Dominik Paul Modest, Frank Tacke, Oliver Haase, Johann Pratschke, Dennis Eurich

**Affiliations:** 1 Department of Surgery, Campus Charité Mitte | Campus Virchow Klinikum, Charité-Universitätsmedizin Berlin, Charité University Medicine Berlin, 13353 Berlin, Germany; 2 Department of Hematology, Oncology and Cancer Immunology, Campus Charité Mitte | Campus Virchow Klinikum, Charité-Universitätsmedizin Berlin, Charité University Medicine Berlin, 13353 Berlin, Germany; 3 Department of Hepatology and Gastroenterology, Campus Charité Mitte | Campus Virchow Klinikum, Charité-Universitätsmedizin Berlin, Charité University Medicine Berlin, 13353 Berlin, Germany

**Keywords:** liver transplantation, de novo malignancy, colorectal carcinoma, immunosuppression

## Abstract

*Background and Objectives:* After liver transplantation (LT), long-term immunosuppression (IS) is essential. IS is associated with de novo malignancies, and the incidence of colorectal cancer (CRC) is increased in LT patients. We assessed course of disease in patients with de novo CRC after LT with focus of IS and impact on survival in a retrospective, single-center study. *Materials and Methods*: All patients diagnosed with CRC after LT between 1988 and 2019 were included. The management of IS regimen following diagnosis and the oncological treatment approach were analyzed: Kaplan–Meier analysis as well as univariate and multivariate analysis were performed. *Results*: A total of 33 out of 2744 patients were diagnosed with CRC after LT. Two groups were identified: patients with restrictive IS management undergoing dose reduction (RIM group, *n* = 20) and those with unaltered regimen (maintenance group, *n* = 13). The groups did not differ in clinical and oncological characteristics. Statistically significant improved survival was found in Kaplan–Meier analysis for patients in the RIM group with 83.46 (8.4–193.1) months in RIM and 24.8 (0.5–298.9) months in the maintenance group (log rank = 0.02) and showed a trend in multivariate cox regression (*p* = 0.054, HR = 14.3, CI = 0.96–213.67). *Conclusions*: Immunosuppressive therapy should be reduced further in patients suffering from CRC after LT in an individualized manner to enable optimal oncological therapy and enable improved survival.

## 1. Introduction

Liver transplantation (LT) is still the only option for various conditions resulting in end-stage liver disease as well as primary malignancies of the liver itself. After LT, life-long or at least long-term immunosuppression (IS) remains standard for the prevention of graft rejection. Here, calcineurin inhibitors (CNI), mycophenolate mofetil (MMF), glucocorticoids (GC) and mammalian target of rapamycin inhibitors (mTORI) are most frequently used, and their fine-tuned regimen is one of the main reasons for the markedly prolonged survival of graft function after LT in the last decades [1]. However, side effects such as chronic kidney injury and neoplasms in the decade-long administration of CNI are well known, and the overall beneficial effects of mTORI are controversial [2,3].

With increasing graft survival, long-term outcomes after LT including comorbidities and complications of IS therapy are gaining more interest. For example, the risk of de novo malignancies (DNM) in patients after LT is significantly elevated with an reported incidence 2- to 3-fold compared with the general population [4,5]. Further, cancer-associated mortality is expected to become the most frequent cause of death in the cohort of LT patients and is already the leading cause of death in the second decade after transplantation [6,7,8].

Colorectal cancer (CRC) is one of the most common malignancies worldwide, and its incidence is elevated after LT [9,10]. The stage-dependent therapeutic regimen is highly standardized and consists of radiotherapy, chemotherapy, surgical resection and optional antibody treatment regarding the individual profile. Compared with the overall population, CRC in LT patients is associated with an increased incidence, comparable with the overall rate of DNMs, and occurrence is reported to be earlier in life [11,12]. Of note, certain underlying diseases leading to LT such as PSC alone or in coincidence with inflammatory bowel diseases (IBD) elevate the risk of development of CRC even further to more than seven times [13,14]. Additionally, non-alcoholic liver disease and hepatocellular carcinoma (HCC) have been associated with increased risk after LT [15]. Reports of outcome after CRC in LT patients are heterogenous. Comparable survival rates have been shown, but also poorer long-term survival in patients after solid organ transplantation [11,16]. However, the handling and especially the clinical impact of modification of IS for LT after the diagnosis of de novo CRC remain unclear, and scientific data are not available, although recommendations have been established recently [17,18].

Previously, we investigated the effect of reduction of immunosuppression in patients suffering from recurrent primary liver malignancies such as hepatocellular carcinoma (HCC) or lung cancer after LT and found an impact on survival for patients with dose reduction upon diagnosis independent of oncological treatment [19,20]. In this study, we investigate patients’ course after diagnosis of de novo CRC after LT with a focus on the impact of immunosuppressive management.

## 2. Patients and Methods

Patients undergoing LT for various conditions at our institution between 1988 and 2020 and with diagnosis of de novo CRC post LT were included in the analysis. Diagnosis of CRC was confirmed by histopathology, and staging was conducted according to guidelines using the classification of the Union for International Cancer Control (UICC) based upon the TNM-classification [21,22]. Oncological regimen was categorized into curative or palliative and best supportive care (BSC).

After LT, all patients were followed up periodically at our outpatient center. Intervals were based on the time after transplantation, ranging from two times a week to every twelve weeks. Here, clinical and laboratory examinations were conducted, and ultrasound-guided, transcostal needle biopsies of the graft were performed according to internal standard protocol at 1, 3, 5, 7, 10 and 13 years and on individual basis thereafter. Routine surveillance via colonoscopy was conducted as recommended by current guidelines, but with intervals of at least five years and intensified surveillance in patients suffering from inflammatory bowel disease (IBD) ranging from once or twice per year to individual intervals as recommended by treating endoscopists [23,24].

To evaluate IS, a score first introduced by Vasudev et al. was used, allowing semiquantitative comparability of different substances (one unit for each daily dose of: prednisone—5 mg, cyclosporine a—100 mg, tacrolimus—2 mg, MMF—500 mg, sirolimus—2 mg) [25]. Cumulative Vasudev score calculated by addition of score over the years and median score were evaluated. Using the approach presented by Rodríguez-Perálvarez et al., impacts of tacrolimus trough levels were analyzed after classification into minimized exposure (<5 ng/mL) and conventional exposure (>5 ng/mL) [26]. Here, mean trough level was calculated (at least one measurement/year) after diagnosis of CRC. For assessment of impact of IS after diagnosis of CRC, management of immune suppressive regimen was grouped in two categories for analysis: (i) maintaining immunosuppression or (ii) new restrictive immunosuppressive management (RIM). RIM was defined when dose reduction or complete discontinuation of IS after diagnosis of cancer was documented. Of note, alteration of mTOR therapy was classified differently: initiation of mTORI without reduction of prior IS was classified as (i) and only if concomitant reduction of other IS (CNI, GC, MMF) was performed were these cases grouped in (ii). Oncological course of patients was followed up by in-hospital data and reports from corresponding institutions, as therapy for LT patients was outlined in an interdisciplinary approach with primary care physicians and oncologists. Thus, data on clinical course as well as laboratory, histological or radiological parameters were extracted from our prospectively maintained database.

Statistical analysis was performed using SPSS Statistics Version 26.0 (IBM Co., Armonk, NY, USA). By its retrospective character, the study design was exploratory. For the testing of statistically significant differences, cross-tables were used for nominal-scaled variables. T-test was applied for continuous, normal-distributed variables. For the testing of non-normally distributed values, the Mann–Whitney U-test or Kruskal–Wallis test were chosen. For the analysis of impact on survival, univariate analysis and Kaplan–Meier analysis were conducted, and log rank tests were calculated. To evaluate effect strength, multivariate and univariate Cox regression models were used, and hazard ratio (HR) and confidence interval (CI) were calculated. Putative relevant variables or confounders for integration in multivariate analysis were identified by clinical experience, such as patients´ characteristics (relevant comorbidities, age, sex) or oncological parameters. A *p*-value of <0.05 was considered significant.

The study was conducted in accordance with the guidelines of the Declaration of Helsinki and was approved by the local ethics committee of our institution (protocol code EA1/255/20; date of approval: 20 October 2020).

## 3. Results

From 2744 patients receiving LT over a 33-year span, 33 patients were identified with de novo colorectal cancer, forming a prevalence of 1.2% in this population. Median time from transplantation to DNM was 12.0 years (0.9–27). Indications for initial LT and overall patient characteristics are displayed in Table 1. Prior to the diagnosis of CRC, immunosuppressants used were CNI (*n* = 28; 84.8%), MMF (*n* = 7; 21.2%), mTORI (*n* = 4; 12.1%) and glucocorticoids (*n* = 1; 3%). A group of 31 (93.9%) patients were diagnosed with colon cancer and two (6.1%) with rectal cancer. Using the UICC criteria, 14 (42.4%) patients were stage I, eight (24.2%) stage II, six (18.2%) stage III and three (9.1%) stage IV at initial diagnosis. Based on staging and patients’ constitution, 32 (97.0%) patients were treated with curative and only one (3.0%) patient with palliative intention. Regimens consisted of oncological resection in 32 (97.0%) cases, chemotherapy in nine (27.3%) and radiotherapy in two (6.1%), with either combination in eight (24.4%) cases based on therapy standards at the specific time. Adjuvant chemotherapy was administered in eight (24.2%) cases, and only one patient received palliative chemotherapy (3.0%). Median survival after diagnosis of de novo CRC was 49.6 (0.5–298.9) months. At the end time of observation, 11 (33.3%) patients had died, and in eight (24.2%), the malignancy was stated as cause of death. In all patients undergoing surgery, histopathology confirmed local R0-resection. We did not find statistical impact of T-stadium or N-classification on survival in Kaplan–Meier analysis, but M1 status was associated with significant shorter survival (log rank 0.001). Kaplan–Meier analysis also revealed the statistical significance of UICC stage on survival after diagnosis with a median of 66.1 (2–129.2) months in stage I, 88.8 (8.4–298.9) months in stage II, 48.8 (0.5–193.1) months in stage III and 36.8 (3.2–55.4) months in stage IV (log rank < 0.01). Regarding decade of diagnosis (1989–1999/2000–2009/2010–2019/2020-today), to account for different oncological therapeutic options, no impact on overall survival after diagnosis was found (log rank = 0.52).

Median IS-score assessed according to Vasudev et al. at time of diagnosis was 2.0 (0.25–6.0) units, and median cumulative IS-score was 30.5 (3.0–87.5). After diagnosis of CRC, 20 (60.6%) patients were identified, where reduction of immunosuppression according to RIM-criteria in response to new malignancy was initiated. Thus, two groups were formed termed RIM and maintenance, respectively. In four patients, IS was withdrawn completely. Mean IS-score did not differ between groups at time of diagnosis with 2.1 (±1.5) units in group RIM and 2.5 (±1.4) units in maintenance group (*p* = 0.5). In RIM-patients, reduction of CNI was initiated in all patients, with relative dosage reduction of 45.0% (0.25–1). Additionally, MMF was reduced in four (20.0%) patients. In four (20.0%) patients, mTORI was introduced into regimen. Immune suppressive regimen prior to the diagnosis of CRC did not differ between the two groups with CNIs as backbone in 19 (95.6%) patients in RIM and in nine (69.3%) patients in the other group. The Wilcoxon test for non-parametric paired variables revealed a dose reduction of IS with statistical significance with an IS-score after prior to diagnosis of 2.1 (±1.5) units and 1.4 (±1.5) after diagnosis of CRC in the RIM-group (*p* < 0.01).

The most frequent indications for LT were alcoholic liver disease (ALD) and primary biliary cholangitis (PBC)/primary sclerosing cholangitis (PSC) in both groups without significant differences (*p* = 0.35). Further, the prevalence of inflammatory bowel disease (IBD) did not differ between groups (*p* = 0.68). Median time to de novo CRC was comparable (RIM: 12.5 (1.0–29.0) years/maintenance: 11.0 (0.9–27.0) years, *p* = 0.44). Furthermore, stage of malignancy using the UICC classification showed no significant difference between groups; most patients were diagnosed with local tumor stages of I/II in 14 (70.0%) patients in the group with restrictive IS management and eight (72.8%) in those with unaltered IS-regimen (*p* = 0.36). Table 1 shows an overview of patient characteristics including oncological parameters. Here, no statistically significant differences between those two groups were found. Additionally, no rejection or loss of graft occurred in the group undergoing further reduction of IS, and thus, no patient received a re-installment of a previous IS-regimen.

**Figure 1:**
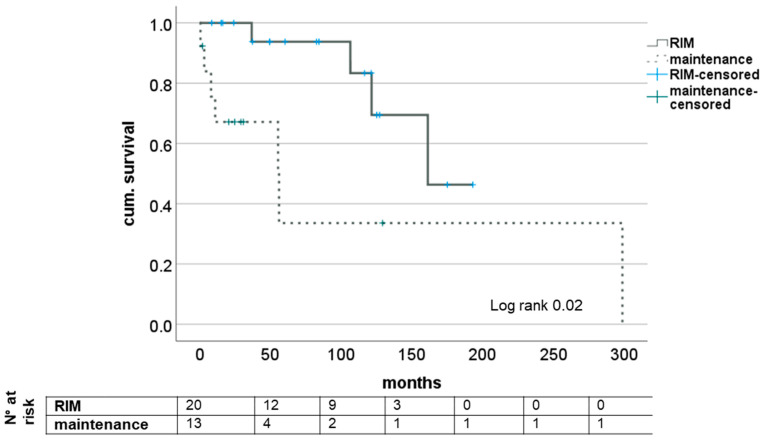
Impact of RIM on survival after diagnosis of de novo CRC after LT. RIM—restrictive immunosuppressive management.

Median survival from initial diagnosis was 83.46 (8.4–193.1) months in the RIM group and 24.8 (0.5–298.9) months in maintenance. At the end of the observation period, four patients (20.0%) had died under restrictive immunosuppression and seven (46.2%) in the group of unaltered IS. Cause of death was CRC in two (20.0%) and five (38.5%). No significance was found in causes of death between groups (*p* = 0.38; see Table 1). Comparison using Kaplan–Meier survival analysis showed statistically significant differences in both short-term and long-term survival (log rank = 0.02); see also Figure 1.

We did not find improved survival after the diagnosis of CRC for the five (15.2%) patients receiving mTORI before compared with those without (log rank 0.13) or those five (15.2%) with mTORI therapy after diagnosis (log rank 0.29).

**Figure 2:**
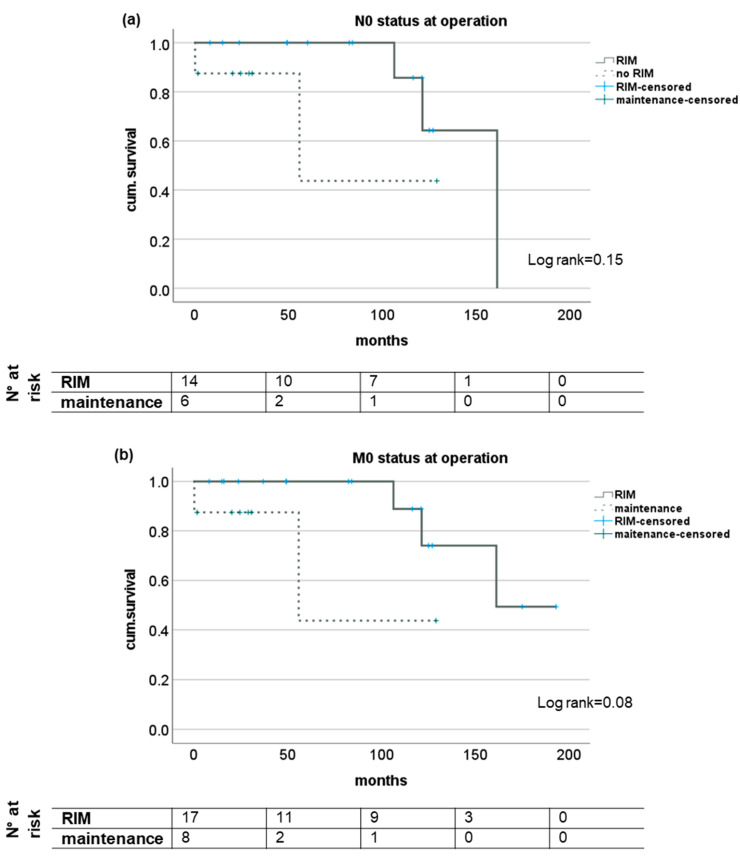

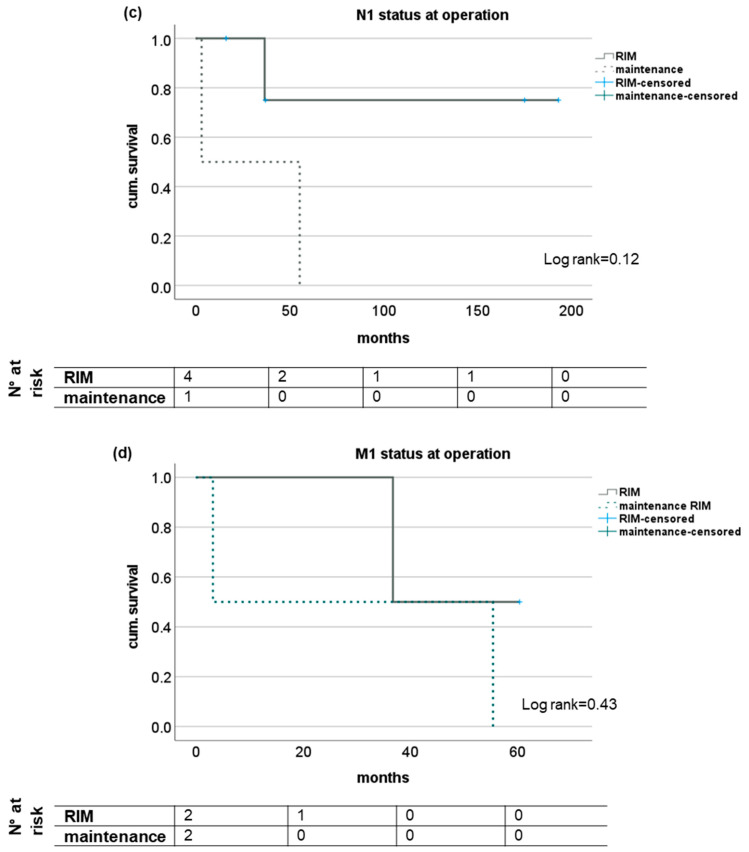
Subgroup analysis of survival of patients with and without RIM dependent on lymph node manifestation or distant metastases at time of diagnosis of CRC after LT. Kaplan-Meier analysis of patients without tumor manifestations in lymph nodes (**a**) or distant metastases (**b**) as well as patients with histological proven tumor manifestation in local lymph nodes (**c**) or distant metastases (**d**) at initial diagnosis of CRC seem to profit from a additional restrictive immunosuppressive regimen upon diagnosis but no statistical significant difference was reached. RIM—restrictive immunosuppressive management.

**Figure 3:**
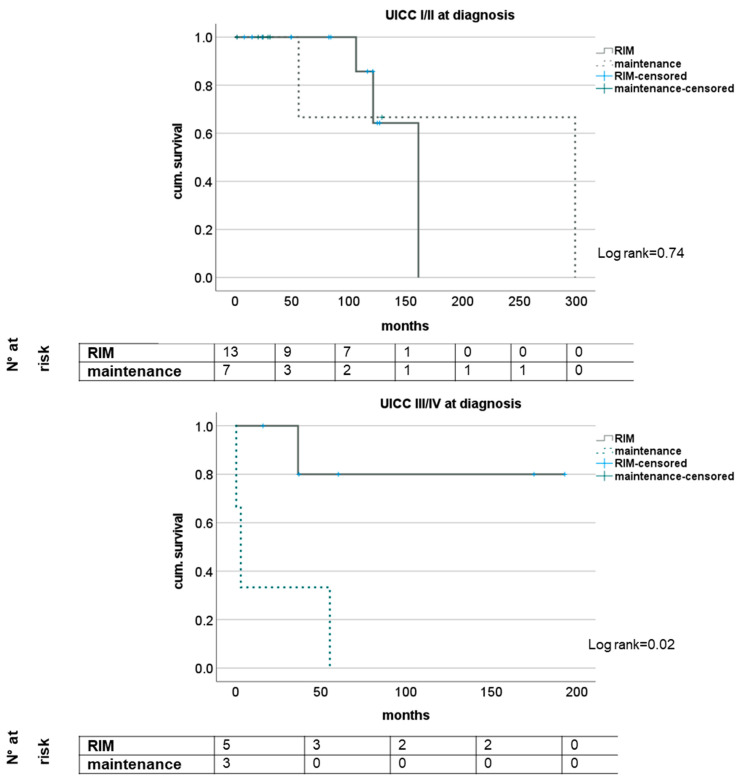
Impact of RIM dependent on tumor stage according to UICC.

The subgroup analysis of patients with regard to N- and M-status showed trends for a survival benefit for patients with RIM but did not reach statistical significance except for short-term survival in patients with M1 status (see Figure 2). Analyzing the survival of patients with or without RIM subgrouped for UICC stage showed no impact in stages I and II but significantly longer survival for patients with UICC stages III and IV when a restrictive immune suppressive regimen after diagnosis of CRC was conducted. Here, median survival was 48.8 (16.2–193.1) months and 3.2 (0.5–55.4) months, respectively (log rank 0.02); see Figure 3.

In multivariate analysis using the clinically important variables of age at tumor diagnosis and preexistent cardiovascular disease and the oncological staging parameters using the TNM classification and RIM, no significant statistical impact on improved overall survival after diagnosis of de novo CRC after LT was found. However, a trend regarding impact of RIM was seen (*p* = 0.054); see also Table 2.

**Figure 4:**
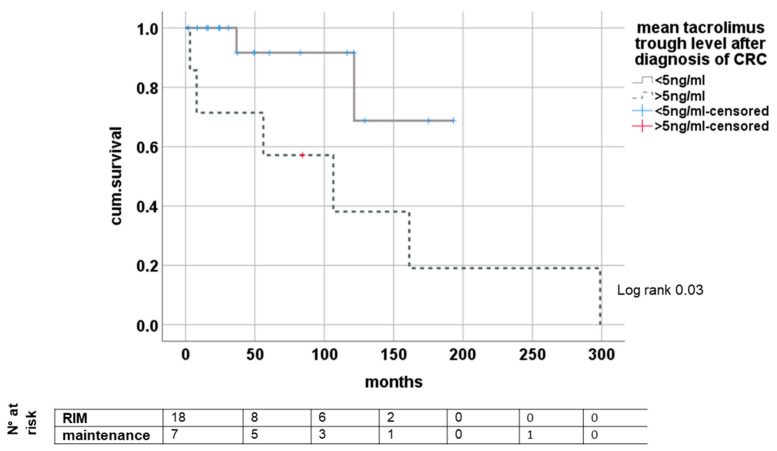
Dependence of survival from tacrolimus trough level after de novo CRC.

Analyzing the impact of tacrolimus trough levels after diagnosis of CRC, we found significantly improved survival in groups with mean trough levels of <5 ng/mL (minimized exposure) and >5 ng/mL (conventional exposure) after diagnosis of de novo CRC, (log rank 0.03); see Figure 4.

## 4. Discussion

In this study, we analyzed the course of patients with CRC after LT. Focus was current IS and its impact on survival, as its influence gains more relevance in recent studies on outcome after LT with regard on long-term survival [26,27,28].

We only found 33 patients out of our cohort of over 2700 patients in a time span of three decades with reported manifestation of CRC, forming a total prevalence of 1.2%, highlighting effective colorectal cancer screening. Studies report an incidence in the general population between 30 and 50/100,000 of new CRC per year in western countries, and an incidence of CRC in LT patients of 4.9% was reported by Altieri et al. [29,30,31]. Due to the life-long follow-up of our patients with high compliance, we do not expect underreporting in our collective but excellent patient adherence to our recommended follow-up examinations that include endoscopies after LT, and thus, many precancerous lesions might have been treated before the manifestation of CRC. This notion was supported by the high fraction of UICC stage I and stage II CRC that formed two thirds of our cohort. Subsequently, curative surgical therapy was available in every patient but one. We found a median occurrence of CRC after LT of 12 years, reflecting the impact of chronic IS and the shift in comorbidities that challenge the aftercare of patients after LT in the long run. Staging-dependent survival rates in our study were comparable with the general population and with LT patients from other reports [13,32]. Staging using the UICC criteria for CRC and the TNM classification demonstrated prognostic value in our cohort, reflecting their importance in decision making [33,34,35,36]. As most patients (all but one) were treated with curative intention with surgical resection, the most relevant impact for survival—surgical resectability—could not be assessed in our study, thus, however, an important potential bias for our study was ruled out in favor of the impact of IS-redesign.

Evaluating the effect of altered handling of IS after diagnosis of CRC, we found the two groups that were formed comparable in all relevant clinical aspects. Thus, impact of RIM could be assessed with validity. Survival analysis revealed positive effects of reducing IS further after de novo malignancy in LT patients, similar to findings for patients suffering from recurrent HCC after LT and in congruence of pathophysiology of administered substances [19,37]. The effect did not reach statistical significance in multivariate analysis, possibly to the very small population. Analyzing the effect of RIM in subgroups, we found impact especially in stages where tumor manifestation was advanced (UICC stages III/IV, M1-status at time of diagnosis). While the utmost importance with highest impact lies undoubtedly within stage-dependent oncological regimen, we hypothesize that the effect of RIM might become evident in cases where overall systemic immune control is overwhelmed, reflecting advanced stages [38,39,40]. As most patients suffering from CRC after LT are found years after the initial transplantation with stable liver function—as indicated in our cohort by the feasibility of major visceral operation—we conclude that RIM should be evaluated as an additional oncological aspect in this special cohort of patients with the aim of complete withdrawal. While early withdrawal has been shown to be of only minor success in certain subsets of patients, long-term discontinuation seems to be more favorable and feasible [41,42,43,44]. However, in an event of a life-threatening disease associated with failed immune response, we deem it mandatory to investigate its practicability in every individual in a step-by-step manner [45,46]. Recently, Colmenero et al. presented guidelines from the ILTS-SETH Consensus Conference regarding the incidence and management of DNMs [17]. While they note the lack of data altogether and the practical absence of prospective studies, their recommendations reflect this study´s findings.

The exact approach to reducing IS in LT patients remains partly unclear and always requires knowledge of the individual patient´s risk profile, comorbidities and tolerance to different substances and their adverse effects [47,48,49]. CNIs remain the most important substance, and all patients undergoing RIM in our study were found with reductions in this drug class. Additionally, we found a tacrolimus through-level-dependent survival difference with beneficial outcome for patients with lower CNI burden. In contrast, mTORI are the only substance of IS where anti-proliferative properties are reported, although its clinical impact remains controversial, and optimal regimen is unclear [50,51,52,53,54,55,56]. We did not find any impact of mTORI on survival, whether administered before or after the diagnosis of CRC, but the number of patients with mTORI was very low. In this regard, using the IS scale proposed by Vasudev et al. might be misguided, as mTORI are weighted equally to CNI, and from our regard, the influence of MMF might be overestimated [25]. However, using the IS scale, a low immunosuppressive burden in the overall cohort was shown, reflecting the modern approach of reducing IS after LT to the tolerable minimum.

Certain limitations of this study have to be addressed. The retrospective, three-decade-spanning character certainly inherited different approaches in post-LT management as well as oncological strategies and therapeutic options that were not explored in depth. Additionally, while the low number of patients reflects the rarity of this special constellation, it especially limited validity for subgroup analysis and also restricts overall statistical analysis. The use of different immune suppressants in over 30 years of LT with diverging focuses (preventing rejection at all cost vs. minimizing adverse side effects for the future) is certainly present in this study and the calculation of IS-score and definition of RIM may be unprecise. However, strategies for CRC have made enormous progress, and regimens including total neoadjuvant concepts for rectal cancer and targeted therapies for metastatic conditions as well as extended concepts for colorectal liver metastases have improved survival for patients immensely [57,58,59,60,61]. We did not evaluate the differentiated oncological strategies, but the distribution of diagnosis of CRC over the decades did not differ between our groups, and thus, possible bias of diverging options even for advanced stages should be ruled out. It has to be acknowledged that while this study further confirms recent recommendations, it inherits the methodical limitations of the few studies published before investigating this issue. Thus, the presented collective can only be regarded as an addition to the growing, but still scarcely existing, body of evidence.

## 5. Conclusions

A remarkable oncological benefit for a restrictive, reflective management of IS upon diagnosis of CRC after LT with significant impact on survival for the individual patients was found in this study. This observation requests timely action from the physician in charge after LT in an individualized manner with close correspondence to treating oncologists as IS reduction can be regarded as an additional oncological measure. To achieve a profound scientific foundation for the reduction of IS in this context, prospective, multi-center data must be acquired in regard to the rarity of occurrence.

## Figures and Tables

**Table 1:** Overall cohort and Group characteristics.

	All Patients (*n* = 33)	RIM (*n* = 20)	no RIM (*n* = 13)	*p* *
Median age at LT years (min-max)	51.0 (14.0–61.0)	50.0 (29–59)	51.0 (14–61)	0.75
sex (%)				0.5
male	19 (57.6)	11 (55)	8 (61.5)	
female	14 (42.2)	9 (45)	5 (38.5)	
Indication for liver transplantation (%)				0.35
ALD	8 (24.2)	5 (25.0)	3 (23.1)	
PBC/PSC	9 (27.3)	6 (30.0)	3 (23.1)	
HCC/CCC	4 (12.1)	4 (20.0)	0 (0)	
viral hepatitis	5 (15. 2)	3 (15.0)	2 (15.4)	
others	7 (21.2)	2 (10.0)	5 (38.4)	
Induction of immunosuppression (%)				0.18
none	11 (33.3)	6 (30.0)	5 (38.5)	
antibodies	16 (48.5)	12 (60.0)	4 (30.8)	
ATG	6 (18.2)	2 (10.0)	4 (30.8)	
Immunosuppression at diagnosis of CRC (%)				0.61
CNI	28 (84.8)	19 (95.0)	9 (69.2)	
MMF	7 (21.2)	4 (20.0)	3 (23.1)	
GC	4 (12.1)	2 (10.0)	2 (15.4)	
mTORI	5 (15.2)	3 (15.0)	2 (15.4)	
combination	6 (18.2)	5 (25.0)	1 (7.7)	
Cardiovascular comorbidities at diagnosis of CRC	24 (72.3)	16 (80.0)	8 (66.7)	0.43
IBD	7 (21.2)	5 (25.0)	2 (15.4)	0.68
BMI at diagnosis of CRC kg/m^2^ (min-max)	23.0 (16–36)	24.9 (16–36)	22.6 (18–27)	0.12
Median age at diagnosis of CRC years (min-max)	60.0 (29–79)	63.0 (36–78)	60.0 (29–79)	0.59
Median time to CRC after LT years (min-max)	12.0 (0.9–27)	12.5 (1.0–29.0)	11.0 (0.9–27.0)	0.44
Decade at time of CRC (%)				0.34
1989–1990	4 (12.1)	1 (5.0)	3 (23.1)	
2000–2009	9 (27.3)	7 (35.0)	2 (15.4)	
2010–2019	18 (54.5)	11 (55.0)	7 (53.8)	
2020-today	2 (6.1)	1 (5.0)	1 (7.7)	
UICC stage				0.36
I	14 (42.4)	10 (50.0)	4 (36.4)	
II	8 (24.2)	4 (20.0)	4 (36.4)	
III	6 (18.2)	5 (20.0)	1 (9.1)	
IV	3 (9.1)	1 (5.0.)	2 (18.2)	
missing	2 (6.1)	-	2 (15.4)	
curative oncological regimen	32 (97.0)	20 (100.0)	12 (92.3)	0.34
Deceased at follow-up	11 (33.3)	4 (20)	7 (53.8)	0.38
cause of death				
CRC	7 (21.2)	2 (10)	5 (38.5)	
cardiovascular	3 (9.1)	1 (5)	2 (15.4)	
other	1 (3.0)	1 (5)	0	

LT—liver transplantation; ALD—alcoholic liver disease; PBC—primary biliary cholangitis; PSC—primary sclerosing cholangitis; HCC-hepatocellular carcinoma; CCC—cholangiocellular carcinoma; ATG—anti-thymocyte globuline; CRC—colorectal cancer; IBD—inflammatory bowel disease; CNI—calcineurine inhibitor; MMF—mycophenolate mofetile; GC—glucocorticoid; mTORI—mammalian target of rapamycin inhibitor; BMI—body mass index; UICC—Union for International Cancer Control. *—comparison of RIM and no RIM.

**Table 2:** Multivariate Regression analysis on impact of survival after diagnosis of CRC after LT.

Parameter	*p*	Hazard Ratio	95% CI	
			Lower	Upper
age at diagnosis of CRC (≤55 vs. >55 years)	0.830	0.708	0.030	16.511
cardiovascular disease (reference: yes)	0.820	1.277	0.156	10.458
T (reference: T1)	0.250	2.349	0.548	10.062
N (reference: N1)	0.354	0.375	0.047	2.982
M (reference: M1)	0.102	6.439	0.690	60.112
RIM (reference: no RIM)	0.054	14.321	0.960	213.671

CRC—colorectal cancer; RIM—restrictive immunosuppressive management.

## Data Availability

The data presented in this study are available on request from the corresponding author. The data are not publicly available due to local ethics committee stipulations and privacy policy of Charité-Universitätsmedizin Berlin.

## Abbreviations

ALD	alcoholic liver disease
BSC	best supportive care
CCA	cholangiocellular carcinoma
CI	confidence interval
CNI	calcineurininhibitor
CRC	colorectal carcinoma
DNM	de novo malignancy
GC	glucocorticoids
HCC	hepatocellular carcinoma
HR	hazard ratio
IBD	inflammatory bowel disease
IS	immunosuppression
LT	liver transplantation
MMF	mycophenolate mofetil
mTORI	mammalian target of rapamycin inhibitor
RIM	restrictive immunosuppressive management
PBC	primary biliary cholangitis
PSC	primary sclerosing cholangitis
UICC	Union for International Cancer Control
